# Prevalence of Anemia and Risk of Adverse Bleeding Effect of Drugs: Implication for Therapy

**DOI:** 10.1155/2012/795439

**Published:** 2012-02-28

**Authors:** Ezekiel Uba Nwose

**Affiliations:** Institute of Clinical Pathology & Medical Research, Nepean Hospital Pathology, Kingswood NSW 2747, Australia

## Abstract

This study aimed to evaluate the progress in reduction of prevalence of anemia in rural Australia. It also investigates the prevalence of hypoviscosity in anaemia with a view to determine the fraction of anaemic patients at risk of drug-inducible exacerbation of anemia. Archived clinical pathology data (*N* = 130, 354) for the period of 1999 to 2008 were utilized. The prevalence of anemia and hypoviscosity was evaluated by working out (i) the number that fell within anemia definition as a percentage of the population and (ii) the number that fell within hypoviscosity definition as a percentage of anemic patients. The prevalence in anemic diabetes and dyslipidaemia was further determined. There was progressive reduction in anemia from 6.1% to 3.2% over the ten years period. Prevalence of anemia is statistically significantly higher in males than in females (*P* < 0.0001), but protein level is lower in anemic females than in anemic males (*P* < 0.01). The results further show that up to 75% of anemic patients may benefit from NSAID or salicylates. This paper highlights differences between genders. It suggests more concerted effort in men's health and speculates a new factor to investigate in women's health.

## 1. Introduction

There are a range of possible complications of anaemia. For example, it can predispose certain therapies to worsen bleeding complications. A perspective in practice that requires further acknowledgement perhaps is the risk of bleeding and/or exacerbated anaemia. Bleeding as a cause of anaemia is very well known and may not be dwelt much upon. When the issue of complications of anaemia comes, the points that easily come to mind are reduced oxygen transport by the blood, which feedforward to tissue hypoxia and fatigue. Others are reduced endurance or exercise capabilities and secondary organ dysfunction such as heart disease [1].

One potential complication and a confounding factor that is omitted in the above list is hypoviscosity syndrome. It is associated with high salicylates level and low platelet count [2] and synonymous to high international normalized ratio that contraindicates anticoagulant and antiplatelet therapies [3]. Thus, it is of particular importance for chronic disease patients such as diabetes and dyslipidaemia. While antiplatelet therapies such as aspirin and aphaeresis such as plasmapheresis are used to reduce blood stasis, “stickiness” or viscosity in order to improve flow rate, hypoviscosity is a state where the flow is quite normal, or nonsteroidal anti-inflammatory drug (NSAID) and antiplatelet could cause the adverse effect of bleeding [4, 5]. Blood hypoviscosity can also be defined as opposite to hyperviscosity syndrome [6]. It results mainly from anaemia and hypoproteinaemia [7–9]. Based on the rationale for therapeutic management of hyperviscosity, the implication is that any patient with hypoviscosity syndrome may not require therapy that may further reduce blood viscosity. For instance, when considering further bleeding risk such as in sickle cell anaemia and systemic lupus erythematosis where salicylates and/or NSAID therapy can cause stomach bleeding and exacerbate the anaemia that is associated with the disease condition [10, 11].

Given the concerns of adverse effects of therapies and the attendant need for evidence of indication or otherwise, it follows that evidence of hypoviscosity translates to contraindication for such therapy. As yet, hypoviscosity is not clinically assessed. Therefore, prevalence of hypoviscosity associated with anaemia is unknown. Anaemia is a condition that has undoubtedly attracted attention long time ago, and it continues to be a thing of health concern especially in women [12]. It is a complication involved in a very wide range of disease [13–16]. It is presumed that knowledge of the prevalence of concurrent hypoviscosity in anemia will benefit future planning and targeted management aimed at avoiding exacerbation. 

*Aim*. This study evaluates the reduction in prevalence of anemia in rural Australia. Further, it investigates the prevalence of anemia and concurrent hypoviscosity in diabetes and dyslipidaemia patients. The latter objective is with a view to speculate the fraction of these patients who may be at risk of bleeding and exacerbated anemia.

## 2. Materials and Methods

This work is part of a Translational Biomedical Science Research initiative. It is supported materially by the Albury South West Pathology, a unit of Western Pathology Cluster of NSW Health Australia. The Ethics Committee of the Area Health Service granted request through the Operations Manager for the use of deidentified data. Ten years deidentified archived clinical pathology data for the period of January 1999 to December 2008 constitutes the database [17]. Selection was limited to those that were concomitantly tested for haematocrit and total proteins from the same phlebotomy collection time. WBV at high shear stress was determined from haematocrit and total proteins as previously published [18].

Specific to the community/population being studied, Albury's South West Pathology reference range for haematocrit were 0.36–0.49 for men and 0.32–0.46 for women, as well as serum total protein being 60–80 g/L for both men and women. These were used to determine that abnormal low WBV is <13.7 CPs for females and <14.2 CPs for males [19]. It was acknowledged that prevalence of anemia depends on the haematocrit thresholds used in the definition [20]. Therefore, the World Health Organization's (WHO) haematocrit thresholds used to define anemia was noted for the different age ranges differed from the rural pathology's cutoff lines (Table 1).

In this study deidentified patients have been used. As the outcome of the study provides no direct or immediate benefit to participant, contact with patients or their clinicians was not made. The implication is that selection of diabetes and dyslipidaemia subjects were limited to those who had hemoglogin-A1c (HbA1c) and lipids test, respectively.

Statistics: First, data for the entire population of patients who visited rural Australian pathology service were sorted into females and males on a yearly basis. Comparative prevalence of anaemia and hypoviscosity between female and male groups was determined and compared. The determination of prevalence followed a simple process of (i) sorting each group by descending level of haematocrit, (ii) highlighting and working out the number that fell within anemia definition as a percentage of the population, (iii) resorting of those highlighted as anemic by descending level of WBV, and (iv) working out the number that fell within hypoviscosity definition as a percentage of anemic patients. The Comparison was performed by Student's *t*-test. 

Secondly, prevalence of anemia and risk of adverse bleeding effect of drugs in diabetes and dyslipidaemia patients were determined. All cases selected as anemic in the 10 years data sets of male and female groups were separately pooled and ranked by age. Children and teenagers were excluded using World Health Organization's age brackets regarding definition of anemia (Table 1). The prevalence of concurrent hypoviscosity in the subgroups was evaluated by repeating the first three steps of the process performed for the general population. Statistical analyses were performed using Microsoft excel analysis tool.

## 3. Results

**Figure 1:**
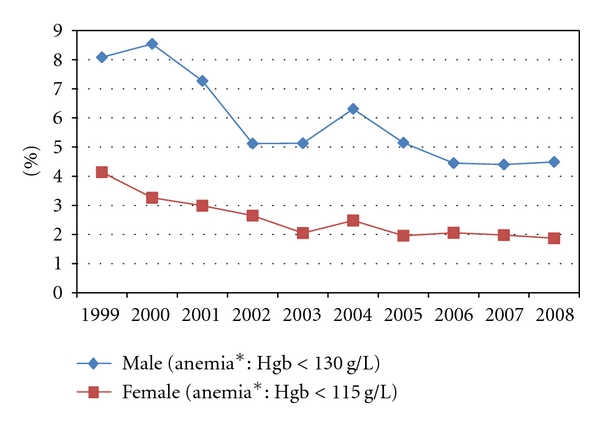
Prevalence of anemia in general population of patients. *Anemia according to regional pathology is adult male with either “Hb < 130 g/L” or “hct < 36%”; adult females “Hb < 115 g/L” or “hct < 32%”.

The female population included “*n* = 117, 746”, out of which 2,796 had anemia. The male population included “*n* = 112, 608”, out of which 6,283 had anemia (Table 2). There is evidence of progressive reduction in anemia over the ten years period from average of 6.1% in 1999 down to 3.2% by 2008 (Figure 1). It is observed that prevalence is statistically significantly higher in males than in females (*P* < 0.0001).

**Figure 2:**
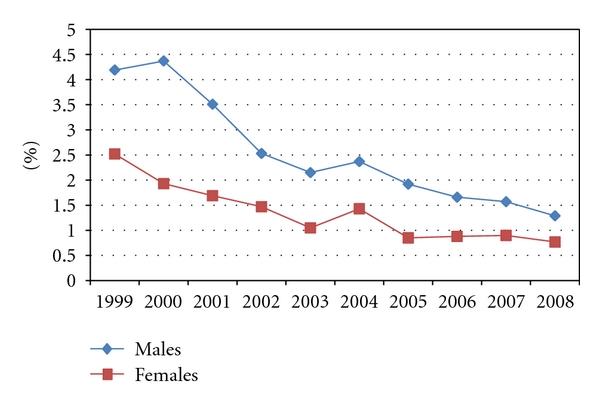
Prevalence of hypoviscosity in general population of patients.

**Figure 3:**
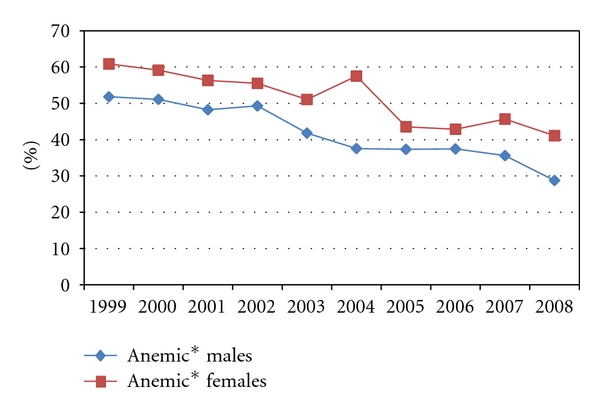
Prevalence of hypoviscosity in anemic patients. *Anemia according to regional pathology is adult male with either “Hb < 130 g/L” or “hct < 36%”; adult females “Hb < 115 g/L” or “hct < 32%”.

Evaluation of hypoviscosity concurrence shows that (i) prevalence of hypoviscosity also progressively decreased over the years from average of 3.4% in 1999 down to 1.0% in 2008 (Table 2). The prevalence is greater in males than in females (Figure 2; *P* < 0.01), but much greater in anemic subpopulations and statistically significantly more in females subpopulation than in males (Figure 3, *P* < 0.01). Further evaluation regarding the average levels of total protein over the ten years period showed it is consistently and statistically significantly lower in the anemic female group compared to the anemic male group (Table 3; *P* < 0.01)

The second analysis was for the special consideration to anemic diabetes and dyslipidaemia patients. The anemic male population included “*n* = 6283”, out of which “*n* = 6, 007” were adults. Others were children and teenagers. Among the adults, 1,367 were tested for lipid profile, of which 130 had dyslipidaemia indicated by total cholesterol >5.5 mmol/L. 571 were tested for hemoglogin-A1c, which was used as indication of ongoing diabetes disease management. 146 were reported as poorly controlled diabetes (HBA1c > 9%). The anemic female population included “*n* = 2, 796”, out of which “*n* = 2, 744” were adults. Others were children and teenagers. Among the adults, 539 were tested for lipid profile, of which 54 had total cholesterol >5.5 mmol/L. 173 were tested for HbA1c, of which 49 were reported as poorly controlled diabetes (HBA1c > 9%).

**Figure 4:**
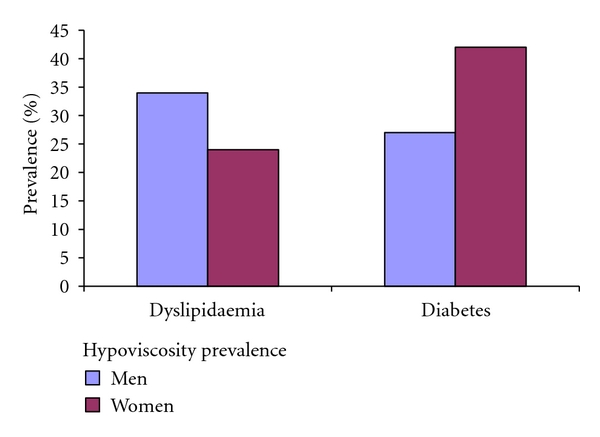
Prevalence of hypoviscosity in anemic diabetes and dyslipidaemia.

On further evaluation for hypoviscosity concurrence with anemia, it is observed that there is at least 24% prevalence of hypoviscosity concurrent with anemia in patients who have diabetes or dyslipidaemia patients (Figure 4).

## 4. Discussion

The first objective of this study was to determine progress in reduction of anemia over a period of ten years using pathology-based evidence. Given that prevalence of anemia depends on the haematocrit thresholds used in the definition [20], it was noted that the World Health Organization thresholds for diagnosis of anemia were different from the reference ranges used by the pathology (Table 1). Therefore, discretion was employed to use pathology's reference values in order to avoid underestimation of the prevalence of anemia in adults.

The results in Figure 1 provide laboratory-based evidence to indicate that there is real progress and success in the efforts towards reducing anemia. If females and males are combined, there is evidence of progressive reduction in anemia over the ten years period from average of 6.1% in 1999 down to 3.2% by 2008 (Figure 1). It also show, however, that the prevalence of anemia is consistently and statistically significantly higher in males than in females (Table 2; *P* < 0.0001). This observation probably reflects the greater efforts to eradicate anemia in women compared to men. It highlights the need for men's health to emulate the attention given to anemia from women [12].

Evaluation of hypoviscosity concurrence shows that the prevalence of hypoviscosity is also progressively decreasing just as anemia is decreasing and that it is more in males than in females (Figure 2; *P* < 0.01). This observation indicates the impact of anemia as a contributing factor to blood viscosity. However, it is interesting to note that percentage prevalence of hypoviscosity is more than ten folds in the anemic subpopulation compared to the general patient population. Further, hypoviscosity is observed to be persistently statistically significantly more in anemic females than in anemic males (Figure 3, *P* < 0.01). That is, although anemia and hypoviscosity are separately more prevalent in the males over the ten years period, the fraction of anemic women who have concurrence of hypoviscosity is significantly more than in males.

The observation presented in Table 3 demonstrates that average total protein levels for the groups are on the side of normalcy and significantly lower in anemic females than in anemic males (Table 3; *P* < 0.01). The salient implication is that hypoproteinaemia is more in anemic females than in males. Hypoproteinaemia is the biochemistry status that would drive a physiological state of hypoviscosity [18], which in turn drives pathological bleeding complication [21].

Perhaps, it is pertinent to differentiate (i) low haematocrit, as the sign that medication is causing loss of blood [22, 23] from (ii) low proteinaemia as a condition that would drive hypoviscosity enroute further bleeding complication.Thus, if given equal number of men and women suffering anemia, more of the anemic women have a confounding condition of hypoproteinaemiathat would make them likely to suffer bleeding complications.

Anemia and hypoproteinaemia have been identified as a symptom complex mainly in cystic fibrosis [24, 25] and requiring medical nutritional supplement for the management [26]. The observation presented in Figure 4 is translated to over 24% percent of anemic diabetes and dyslipidaemia patients having confounding factor that predisposes them to risk of bleeding, if treated with antiplatelet or NSAID. The clinical importance is that it reflects the fraction of anemic patients who may require consideration for therapies that would not exacerbate existing anemia condition.

Perhaps, it is pertinent to mention that there are alternatives to drugs. A case has been reported of woman who complained of bleeding bruises as side effect to aspirin. Over a long time, her General Practitioner did not receive any pathology report that contraindicates the therapy. The patient attended a diabetes research screening clinic where WBV was tested and identified to be in the abnormal low range. Based on the WBV result, patient resorted to nutrition and physical activities as alternative therapeutic measures [27].

Neither WBV nor hypoviscosity syndrome is as yet routinely assessed in clinical practice. Therefore, this report is speculative and suggestive. The premise is strong that blood is *less sticky*, antiplatelet therapy could cause too much bleeding and exacerbate any existing anemia [4, 21]. The novelty of this report is in threefolds: (i) pathology-based evidence that a substantial number of anemic patients have confounding factor (hypoviscosity driven by proteinaemia) that could predispose them to risk of bleeding and exacerbation of anemia, (ii) in addition haematocrit, protein level from routine liver function test can be utilized to determine concurrence of hypoviscosity to rule of risk of exacerbating anemia, and (iii) previous report indicated that 97.5% of cases investigated for chronic diseases could benefit from antiplatelet medication [28], but this study indicates only up to 75% of anemic patients.

## 5. Conclusion

This paper indicates that hypoviscosity driven by low proteinaemia is highly prevalent in anemia. The speculation and suggestion here are that laboratory monitoring of stasis using proteinaemia and/or WBV measures should provide evidence-based pathology for risk of exacerbating anemia in anemic patients.

## Figures and Tables

**Table 1:** Hematocrit thresholds for definition of anemia.

Age & gender	Rural pathology	WHO
Children <5 years	34	33
Children 5–<12 years	35	35
Children 12–15 years	36	36
Men >15 years	36	39
Women >15 years	32	36
Pregnant Women	32	33

**Table 2:** Numbers of anaemia with confounding hypoviscosity among females and males.

	Females			Males		
	*N*	Anaemic	Hypo-WBV	*N*	Anaemic	Hypo-WBV
1999	4449	184	112	4418	358	185
2000	9374	306	181	8980	767	392
2001	11089	332	187	10546	767	370
2002	11291	299	166	11321	580	286
2003	11454	235	120	10717	550	230
2004	12334	306	176	11653	735	276
2005	12677	248	108	11984	616	230
2006	14278	294	126	13338	593	222
2007	15175	300	137	14610	644	229
2008	15625	292	120	15041	675	194

WBV: whole blood viscosity.

**Table:** (a) Overall descriptive statistics

	Females	Males
Mean (g/L)	62	64
Median (g/L)	62	65
SD	11	10
Minimum (g/L)	23	24
Maximum (g/L)	182	130
Count	2796	6283

**Table:** (b) Mean protein levels (g/L) by year

Year	Females	Males
1999	61	62
2000	59	62
2001	61	63
2002	60	62
2003	63	63
2004	61	64
2005	63	65
2006	64	64
2007	63	65
2008	64	66
